# Correlates of Meal Skipping in Community Dwelling Older Adults: A Cross-Sectional Study

**DOI:** 10.1007/s12603-023-1884-2

**Published:** 2023

**Authors:** H. Wild, D. Gasevic, R.L. Woods, J. Ryan, M. Berk, R. Wolfe, J. McNeil, A.J. Owen

**Affiliations:** 1. School of Public Health and Preventive Medicine, Monash University, 553 St Kilda Melbourne 3004, VIC, Australia; 2. Centre for Global Health, Usher Institute, Teviot Place, Edinburgh EH8 9AG, UK; 3. The Institute for Mental and Physical Health and Clinical Translation (IMPACT), School of Medicine, Deakin University, and Barwon Health, Geelong, Victoria, Australia; 4. Orygen, the National Centre of Excellence in Youth Health, and the Florey Institute for Neuroscience and Mental Health, Department of Psychiatry, University of Melbourne, Melbourne, Victoria, Australia

**Keywords:** Meal skipping, older adults, nutrition, positive ageing

## Abstract

In this cross-sectional analysis of 10,071 community dwelling adults aged ≥70 years, we examined factors associated with meal skipping (self-reported) using multivariable logistic regression. Prevalence of meal skipping in this study was 19.5%. The adjusted odds (aOR [95%CI]) of meal skipping were lower in those 85+ years (vs. 70-74.9 years, 0.56 [0.45-0.70]), and in those in regional areas (vs. urban area, 0.81 [0.72-0.92]). Higher odds of meal skipping were observed for those living alone (vs. living with someone, 1.84 [1.64-2.05]), current smokers (vs. non-smokers, 2.07 [1.54-2.80]), consumers of high amounts of alcohol (vs. abstainers 1.93 [1.35-2.75]), those with poor oral health (vs. excellent oral health, 1.71 [1.07 −2.73]) diabetes (vs. not 1.26 [1.06-1.50]), or frailty (vs. not, 1.63 [1.09-2.43]). This study identified socio-demographic, social, behavioural and biomedical correlates of meal skipping in later life, which may assist in targeting interventions to address meal skipping.

## Introduction

Nutrition-related diseases are major contributors to disability and lowered quality of life in older age (1), and nutrition survey data consistently suggest that people over the age of 65 years do not meet their daily recommended nutritional or energy requirements (2). Nutrient and energy deficiency in later life can increase the risk and the severity of age related chronic disease (3).

It is accepted that there is a relationship between the number of meals consumed per day and overall dietary quality in those over the age of 65 years (4, 5). It is also common for dietary intake to change in quantity and quality in later life, with dietary behaviours such as meal skipping cited as influential in the development of late-life malnutrition (4).

Meal skipping is defined as the omission of one of the traditional daily meals, breakfast, lunch, or dinner (6, 7). A recent systematic review (8) on the prevalence and correlates of meal skipping in community dwelling older adults, reported variability in the proportion of adults who consumed less than 3 meals daily, ranging from 2.1 to 61%. Advancing age, male gender, and social exclusion were reported as significant correlates of meal skipping though the evidence base is limited by non-contemporary and heterogeneous studies (8) Sustaining nutritional status in community dwelling older adults is important to lower the risk of age-related disease, hospitalisation and institutionalisation (9) and to maintain independent living (4). The current evidence base on food behaviours in older adults is predominantly centred on institutionalised people, with limited research on those living independently in the community (9).

Therefore, the aim of this study is to determine the prevalence and socioecological factors associated with meal skipping in a large cohort of community dwelling adults aged 70 years and over.

## Methods

### Study population

The ASPirin in Reducing Events in the Elderly (ASPREE) study was a multi-centre randomised placebo-controlled trial of low dose aspirin conducted in Australia and the United States (U.S.) between 2010 and 2017. The Australian ASPREE cohort included 16,703 healthy, community dwelling, participants aged 70 years or above who were, at baseline, free from persistent physical disability, dementia, cardiovascular disease or chronic/serious disease that was likely to be fatal within 5 years (10). Details of the inclusion and exclusion criteria and methods have been previously described (10, 11). The ASPREE Longitudinal Study of Older Persons (ALSOP) is a longitudinal cohort study, comprised of 14,892 Australian participants from ASPREE (10). For this study, participants with missing data on exposure (meal skipping) (n=2,466) and independent variables of interest (n=2,355) were removed from the primary analysis dataset. (See Supplementary Figure 1).

### Meal skipping

Self-reported dietary intake, including the frequency of meal skipping each week, was ascertained via the year-3 ALSOP medical questionnaire (10). Participants were asked “How often do you miss meals?”, and they were able to choose from the following responses: “never/rarely, once a week or less, several times a week, everyday”. In line with the available evidence (6), and due to small distribution of meal skipping across all categories of the meal skipping variable, we created a variable that assessed whether meal skipping was present or not (yes/no). We classified responses “once a week or less”, “several times a week” or “every day” as “yes”, and “never/rarely” as “no”.

### Correlates

The selection of potential correlates was informed by previous research into dietary patterning in older adults (1). These factor correlates were grouped in the following categories: 1) economic and demographic, 2) health behaviours 3) biomedical, 4) social, 5) psychological. The majority of measures were assessed at 3-year ASPREE/ALSOP follow-up interviews , with the exception of years of education, area-level socioeconomic status using the Index of Relative Social Advantage and Disadvantage (IRSAD) (12) and presence of hypertension, polypharmacy, frailty and diabetes which were taken at baseline in the ASPREE trial. The Centre for Epidemiological Studies Depression (CES-D) scale (13) was used to assess level of depressive symptoms and baseline quality of life was assessed using the SF-12 questionnaire, condensed to physical (PCS) and mental component scores (MCS) with higher PCS and MCS scores indicating higher physical and mental quality of life. The mean PCS and MCS were calculated for the study population, and participants were categorised into above or below the average score (14). Behavioural factors were assessed via self-reported questionnaires. In the year-3 ASPREE follow up interviews, participants reported their smoking status, daily consumption and daily alcohol intake. The year-3 ALSOP medical questionnaire asked participants to rate their difficulty performing tasks such as reading labels. Biomedical factors were assessed via combination of clinical and self-reported information at baseline (polypharmacy, hypertension, diabetes and frailty) and at year-3 by the ALSOP medical questionnaire (BMI, oral health status, saliva levels and pain frequency). Living status was assessed by the year-3 ALSOP social questionnaire, participants were asked to report who they lived with. (For additional detail on how correlates were measured please see Supplementary Table 1).

### Statistical analysis

Participant characteristics were presented as counts and percentages based on the meal skipping status. Differences in study characteristics between participants who rarely or never skip meals compared to those who skip meals were assessed using Chi-squared tests and two-way T-tests. The association between socio-ecological factors and meal skipping was assessed using multivariable logistic regression where all potential correlates were mutually adjusted. Odds ratios and 95% confidence intervals (CI) were reported. BMI is a recognised correlate of meal skipping in older adults (4, 15, 16) however, substantial missing data were observed for BMI (n=1,194); hence, the variable was removed from the primary analysis. Instead, a multivariable binary logistic regression sensitivity analysis model that included BMI was undertaken (n=8,877). Similarly, depression correlates with appetite loss 17 in older age, however, a considerable amount of data was also missing for the depression variable (n= 1,021), and a further sensitivity analysis included all variables in the primary model and the CESD-10 scores (n=9050).

We performed correlation analyses between the hypothesized correlates of meal skipping. The highest correlation coefficient reported was between PCS and pain frequency variables (r=0.33) suggesting no multi-collinearity in the data. All statistical analysis was performed in Stata statistical software version 17.0 (StataCorp LLC, College Station, Texas; www.stata.com) (18).

## Results

### Participant characteristics

There were 10,071 participants (54% were female) included in this study with mean age of 77.9 years at the time of meal skipping assessment. A majority of participants lived in major metropolitan areas, lived with others, had ≤12 years of education, lived in high socioeconomic areas (29.9%) and were non-smokers (97.9%) (Table 1). Participant characteristics were compared between participants with missing data (n=2,466) and those without (n=10,071) with no substantial differences observed.

### Prevalence of meal skipping

Four in five (80.5%) participants reported never or rarely skipping meals, 14.9% reported skipping meals once a week, 3.8% reporting skipping meals several times per week and less than 1% reporting skipping meals daily. The total prevalence of meal skipping in this study population of adults aged 70 years and over was 19.5%.

Compared to people who never or rarely skipped meals, those who reported any meal skipping were younger on average, had more than 12 years of education, reside in inner city locations, and have poorer oral health. People who skipped meals were also more likely to live alone, to consume over 3 alcoholic drinks daily, be current smokers and be diagnosed with diabetes and be classified as frail. (Table 1)

### Correlates of Meal Skipping

**Figure 1.**
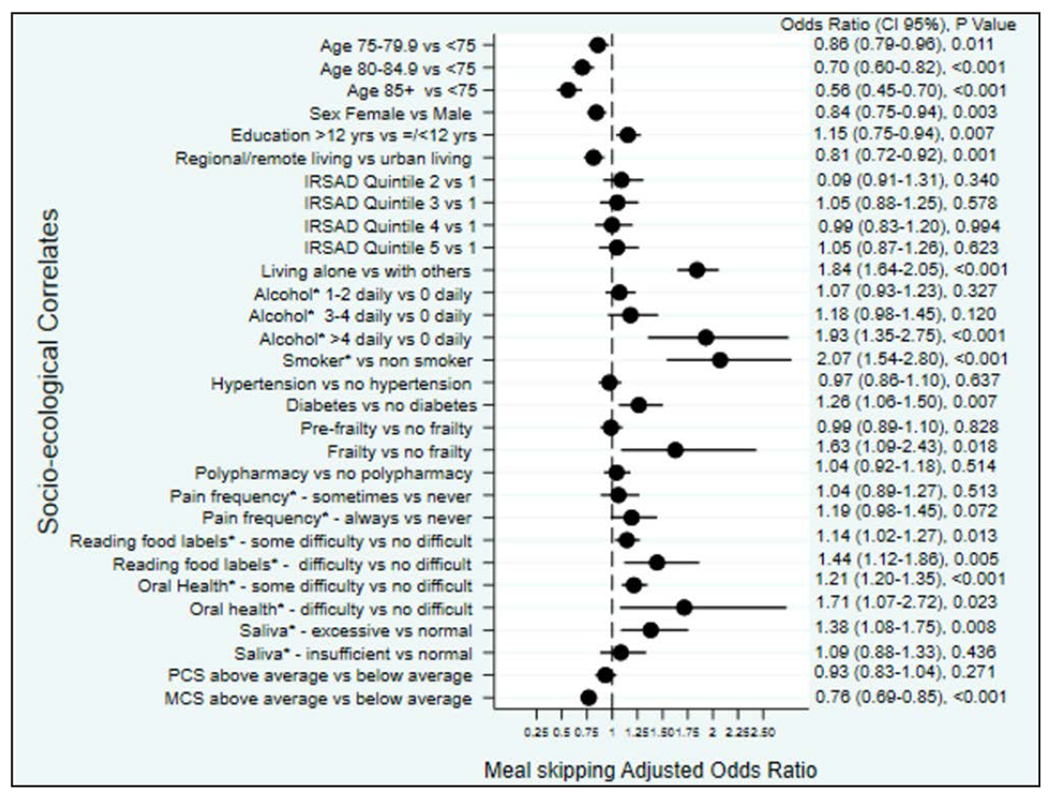
Forrest Plot: The association of demographic, social, behavioural, biomedical, and psychological factors with meal skipping in adults aged 70 years and over: the results of the multivariable binary logistic regression analyses OR= odds ratio, CI=confidence interval, IRSAD=index of Relative socioeconomic Advantage and Disadvantage 12 *= self-reported; PCS= Physical Component Scores of the SF-12 questionnaire; MCS= Mental Component Score of the SF-12 questionnaire; Hypertension = systolic blood pressure ≥140mmHg and diastolic blood pressure ≥90mmHg or taking blood pressure-lowering medication (11, 29); Diabetes mellitus = self-reported, taking medication for diabetes, or a fasting glucose ≥126mg/dL (11, 29); Frailty is based on modified Fried frailty criteria of low body weight, low grip strength, exhaustion, low physical activity and slow walking speed. 29, 30 Those classified as pre-frail met one or two of the Fried criteria, and those classified as frail met 3 or more criteria. (29, 30); Polypharmacy = self-reported, or confirmed in medical record, of 5 or more prescription medications

The odds of meal skipping were lower among adults aged 85 years and over (vs. 70-74.9 years OR [95%CI], 0.56 [0.45-0.70]), in women (vs. men 0.84 [0.75-0.94]), in those living in regional and remote areas (vs. those living in major cities, 0.81 [0.72-0.92]) and in those with above average MCS (vs. those with below average MCS 0.76 [0.69-85]). In contrast, odds of any meal skipping were greater among older adults living alone (vs. with others 1.84 [1.64-2.05]), those who smoked (vs. non-smokers 2.07 [1.54-2.80]), those who consumed more than 4 alcoholic drinks a day (vs. those who abstained 1.93 [1.35-2.75]), and individuals reporting more than 12 years of formal education (vs. those reporting 12 years or less of formal education 1.15 [1.04-1.28]). People diagnosed with diabetes had higher odds of meal skipping (vs. those without diabetes 1.26 [1.06-1.50]). Higher odds of meal skipping were also observed among older adults with frailty (vs. those without 1.63 [1.09-2.43]). (Figure 1 and Supplementary Table 2)

With regard to oral health, older adults who reported good/fair oral health or poor oral health had higher odds of meal skipping (vs. those who reported excellent oral health 1.21 [1.10-1.35]; 1.71 [1.07 −2.73], respectively). Odds of meal skipping were also higher among people who reported having difficulty reading food labels (vs. those who did not report difficulty 1.44 [1.12-1.86]). (Figure 1 and Supplementary Table 2)

### Sensitivity analyses

Results were similar after the additional inclusion of BMI in the regression model. No association was observed between meal skipping and BMI. (Supplementary Table 3)

After inclusion of depressive symptoms (CESD-10 overall score) to the primary model, the results indicated that the odds of meal skipping were 26% (1.26 [1.11-1.43]) greater for those who experienced mild depressive symptoms, and 60% (1.6 [1.36-1.90]) greater for those who reported moderate to severe depressive symptoms compared to those who did not report depressive symptoms (Supplementary Table 3). When compared to the primary analysis, the association between female sex and meal skipping did not maintain its significance with meal skipping in this model (0.99 [0.88-1.11], 0.898). The association between smoking and meal skipping was strengthened by the addition of depression to the multivariate model, with greater odds of meal skipping reported for current smokers (compared to non-smokers 2.28 [1.70-3.65]) when compared with the primary model (2.07 (1.54-2.80), <0.001). (Supplementary Table 3)

## Discussion

We report a wide-ranging examination on the prevalence and the sociodemographic, behavioural, biomedical, psychological and social factors associated with meal skipping in more than 10,000 community-dwelling individuals aged 70 years and over. Among the strongest correlates, we observed that the oldest age group (85+), women and those with above average mental health component scores, were less likely to skip meals; while those who smoked, consumed more than 4 alcoholic drinks daily, lived alone and reported poor oral health were more likely to skip meals. These results are important, as they point to correlates associated with meal skipping, that if addressed may help reduce nutritional deficiency in later life.

The majority of participants in this cohort rarely or never skipped meals. The observed meal skipping prevalence of 19.5% is consistent with similar research in Korean older adults, which noted that 20.9% of participants indicated some level of meal skipping (5). It is also similar to recent statistics on breakfast skipping in adults, whereby King et al (19) reported that 17.1% of adults skipped breakfast. However, it differs from the prevalence observed in children (10%) (7), adolescents (29.9%) (20) and younger adults (10%) (21) highlighting the need for age specific research on important dietary behaviours, such as meal skipping.

We observed that adults aged 85 years and over were less likely to skip meals compared to those aged 70 to 74.9 years. While demonstrating a similar pattern of consumption across age groups to that reported in the current literature on meal skipping in older adults (5), our findings were more pronounced.

Our results indicate that women were less likely to skip meals compared to men, a result that is consistent with the current evidence (4, 16). Previous research (1) notes that nutritional vulnerabilities are increased for men, especially after the death of a spouse, due to a lack of nutritional knowledge and food preparation skills. Interestingly, the results of our sensitivity analysis demonstrated that further adjustment for depressive symptoms weakened the association between sex and meal skipping. This may be due to the increased likelihood of Australian women to report depressive symptoms compared to men (22); a disparity that is also likely to be exacerbated by generational factors associated with the age of this cohort (22). Previous research on the ASPREE cohort (23) has demonstrated a higher prevalence of women reporting depressive symptoms (CESD-10 overall score 8 or above) compared to men, consistent with findings international cohorts of older adults (24).

In ASPREE participants, the odds of meal skipping were greater among those who smoked and those who consumed higher levels of alcohol compared to those who did not. Smoking is frequently associated with meal skipping in older adults, 5, 15, 16 as well as in younger adults (6, 21). Conversely, findings on the association between alcohol consumption and meal skipping are mixed, with studies in older adults demonstrating both positive (16), negative (5) or no (15) association between alcohol consumption and meal skipping.

This study observed that those who reported living alone had higher odds of meal skipping than people who reported living with others. These results are consistent with the current body of evidence which highlights living alone as a common factor associated with meal skipping among older adults (4, 5, 15).

This study also highlighted the influence of oral health on meal skipping. Changes in dietary intake in later life as a result of poor oral health have been reported in the literature with Gu et al. (25) noting a shift toward a more refined western diet in those over 80 years with difficulty chewing. When considering oral health within the Australian context, it is important to acknowledge that dental care was not, at the time of this study, provided to adults as part of the publicly funded universal healthcare system. This is likely to have increased the influence of the socioeconomic gradient on oral health outcomes (26). The ALSOP cohort is skewed toward a higher socio-economic status (based on area levels of advantage and disadvantage) and education level (higher level of tertiary education 23% vs 2.4%) compared to the general Australian population over the age of 70 years (10). As a result, these participants are likely to have increased access to the financial resources required for suitable dental care compared to their counterparts in the general Australian population. As such, the influence of oral health on meal skipping may be more pronounced within the general community than within this cohort. Food insecurity affects 1 in 50 adults older than 65 years in Australia and can impact food intake (27). Socioeconomic factors have a been shown to have a significant impact on food security status for older Australians (27). Due to the noted differences in socioeconomic status between the ALSOP cohort and the general community, the influence of food insecurity on meal skipping is likely underrepresented in this study.

The results of our sensitivity analysis highlighted a positive association between severity of depressive symptoms and odds of meal skipping. This is consistent with evidence on meal skipping in older adults (5). Depression is often characterised by lowered appetite (24), interestingly, the results of this sensitivity analysis demonstrated that depression strengthened the association between smoking and meal skipping, a behaviour that can also influence appetite and food intake (28). Further research may be required to understand the association between variations in symptoms of depression and meal skipping.

Acknowledging the interrelationship of many of the psychological, social, economic, behavioural and biomedical correlates highlighted in our results is important. It will be essential for future research on meal skipping to further examine the ways in which these factors accumulate across the lifespan, and how their interaction in older age influences health behaviours, and alters the nature and severity of disease risk. The diverse implications of social and psychological factors in older age emphasise the importance of tailored psycho-social services that meet the specific needs of community dwelling older adults. It is likely that many of the protective factors for health and wellbeing in later life are established in younger years, further highlighting the importance of a lifespan focus in health research to identify and manage the socio-ecological factors associated with disease development in later life (8).

The strengths of this research include use of a large, well-characterised sample of older adults, high response rates to questionnaires and strong inclusion and exclusion criteria. 10 There are also several limitations. This is a cross sectional study, so we cannot make any conclusions about the direction of the relationship between the socio-ecological correlates and meal skipping. The use of secondary data also limits the type of information available within the analysis, as such the influence of broader socio-political and environmental correlates on meal skipping could not be assessed. This issue was also noted by Pendergast et al. (21) in their study on the socio-ecological correlates of meal skipping in younger adults, and potentially highlights the need for nutritional research to more consistently consider and explore the influence of the socio-political and environmental context on dietary behaviour. The Year 3 ALSOP medical questionnaire did not define meal in its question on meal skipping, as such no detail on which meal was skipped is provided, and differing cultural and social definitions of meal may increase the risk of misunderstanding and misclassification. We condensed the meal skipping variable from four categories (never/rarely, once a week or less, several times a week, everyday) to two (yes/no) and while this mirrored other research on this topic (6), it may have reduced our ability to discuss differing severities of meal skipping. Future research may benefit from more robust data, allowing for a more granular understanding of the differing frequencies of meal skipping, and the specific meals skipped in older adults. The ASPREE/ALSOP cohort may possibly be a healthier and wealthier subset of the community, as a result of the ASPREE inclusion criteria and their interest in participating in a long-term study of healthy ageing (10, 11). However, it should be noted that by the time the self-reported meal skipping measure was assessed (3 years after enrolment into the study), a number of participants had developed chronic disease (29). This ‘healthy cohort’ effect, compared to the general population, is a factor to consider in the interpretation of the study results, and we advise caution when generalising these findings to population with a lower area level socioeconomic status. A larger body of longitudinal research on meal skipping across the lifespan may be necessary to better determine the influence of age on eating behaviour including meal frequency. Finally, for some variables year 3 data were not available, so baseline derived variables were utilised. This potentially increases the risk of misclassification of participants who may have potentially being diagnosed with hypertension, diabetes, and frailty post baseline.

## Conclusion

In this study on more than 10,000 adults aged 70 years and over, we observed that the prevalence of meal skipping was 19.5%. Numerous factors associated with meal skipping were identified, including living circumstance, sex, alcohol intake and smoking status. To better understand the influence of these factors on dietary behaviours such as meal skipping, it will be important for future research to focus on their influence and interaction across the lifespan, and the multiplicity of risk associated with their interaction, and to what extent these factors operate independently or converge on a common mediator such as depression. The results of these analyses, and the identification of key correlates of meal skipping in older adults will be important to inform further investigation on the impact of meal skipping on health outcomes in this cohort.

## Supplementary Material

1

2

## Figures and Tables

**Table 1.** Characteristics of adults aged 70 years and over stratified by meal skipping status

Variable	Total N=10,071	Meal skipping Rarely/Never n=8,105	Meal skipping Present n=1,996	P value
Sex (n)%				0.558
Male	4597 (45.6)	3688 (45.5)	909 (46.2)	
Female	5474 (54.4)	4417 (54.5)	1057 (53.8)	
Age, Mean (SE)	77.96 (0.04)	78.05 (0.46)	77.62 (0.09)	<0.001
Age n (%)				0.002
70-74.99	3018 (30.0)	2373 (29.3)	645 (32.8)	
75-79.99	4428 (44.0)	3564 (43.9)	864 (43.9)	
80-84.99	1856 (18.4)	1523 (18.8)	333 (16.9)	
85+	769 (7.6)	645 (8.0)	124 (6.4)	
Living Status n (%)				<0.001
With others	6771 (67.2)	5633 (69.50)	1138 (57.9)	
Alone	330 (32.8)	2472 (30.50)	828 (42.1)	
Education n (%)				0.004
=/<12 years	5805 (57.6)	4729 (58.4)	1076 (54.7)	
>12 years	4266 (42.4)	3376 (41.6)	890 (45.3)	
Rurality n (%)				<0.001
Inner Cities	5444 (54.1)	4299 (53.0)	1145 (58.2)	
Regional	4627 (45.9)	3806 (47.0)	821 (41.8)	
IRSAD n (%)				0.286
1 (most disadvantaged)	1530 (15.2)	1252 (15.4)	278 (14.8)	
2	1682 (16.7)	1359 (16.8)	323 (16.2)	
3	1852 (18.4)	1498 (18.5)	354 (18.0)	
4	1990 (19.8)	1605 (19.8)	385 (19.4)	
5 (least disadvantaged)	3017 (29.9)	2391 (29.5)	626 (31.6)	
Alcohol drinks per day n (%)				<0.001
0	1742 (17.3)	1431 (17.7)	331 (15.8)	
1-2	7214 (71.6)	5817 (71.8)	1397 (71.1)	
3-4	938 (9.3)	737 (9.0)	201 (10.2)	
Over 4	177 (1.8)	120 (1.5)	57 (2.9)	
Smoke n (%)				<0.001
Past/Non-smokers	9860 (97.9)	7970 (98.3)	1890 (96.1)	
Smokers	211 (2.1)	135 (1.7)	76 (3.9)	
Hypertension n (%)				0.827
No	2639 (26.2)	2120 (26.2)	519 (26.4)	
Yes	7432 (73.8)	5985 (73.8)	1447 (73.6)	
Diabetes n (%)				0.002
No	9183 (91.2)	7426 (91.6)	1757 (89.4)	
yes	888 (8.8)	679 (8.4)	209 (10.6)	
Frailty n (%)				0.003
Not frail	6,600 (65.5)	5335 (65.8)	1265 (64.3)	
Pre-frail	3347 (33.2)	2685 (33.1)	662 (33.7)	
Frail	124 (1.3)	85 (1.1)	39 (2.0)	
Polypharmacy n (%)				0.005
No	7694 (74.4)	6240 (77.0)	1454 (74.0)	
Yes	2377 (23.6)	1865 (23.0)	512 (26.0)	
Pain n (%)				<0.001
Never	1075 (10.7)	895 (11.0)	180 (9.2)	
Rarely sometimes	5520 (54.8)	4490 (55.4)	1030 (52.4)	
Often/always	3476 (34.5)	2720 (33.6)	756 (38.4)	
Reading Labels n (%)				<0.001
No difficulty	4167 (41.5)	3441 (42.5)	726 (36.9)	
Some difficulty	5533 (54.9)	4392 (54.2)	1141 (58.1)	
Difficulty	371 (3.7)	272 (3.3)	99 (5.0)	
PCS Score Mean (SE)	48.90 (0.08)	49.02 (0.09)	48.32 (0.20)	0.001
MCS Score Mean (SE)	56.13 (0.06)	56.40 (0.07)	55.00 (0.16)	<0.001
Oral Health n (%)				<0.001
Excellent	5646 (56.1)	4664 (57.6)	982 (49.9)	
Good	4335 (43.0)	3380 (41.8)	955 (48.6)	
Poor	90 (0.9)	61 (0.6)	29 (1.5)	
Saliva status n (%)				<0.001
Right amount	9102 (90.4)	7377 (91.0)	1725 (87.7)	
Too much	382 (3.8)	279 (3.5)	103 (5.2)	
Too little	587 (5.8)	449 (5.5)	138 (7.1)	
Depression* n (%)				<0.001
None	3826 (42.3)	3232 (44.2)	594 (34.3)	
Mild	3793 (41.9)	3031 (41.4)	762 (44.0)	
Moderate/Severe	1431 (15.8)	1054 (14.4)	377 (21.7)	

P value = difference between groups, SE=standard error, IRSAD=Index of Relative Socioeconomic Advantage and Disadvantage 12, PCS= Physical component scores of the SF-12 questionnaire, MCS= Mental Component Score of the SF-12 questionnaire,

* Depression scores: none= CESD-10 score less than 2, Mild= CESD-10 Score between 3 and 8, Moderate/Severe= CESD-10 score above 8

## Data Availability

The datasets generated and/or analysed during the current study are not publicly available due data being part of a large ongoing observational cohort study with a rigorous process to access data. The datasets generated and/or analysed during the current study are available in the ASPREE clinical trial data resource repository, https://aspree.org/aus/researchers/. The ASPREE clinical trial data resource is managed in partnership with the US, in the Australian ASPREE National Coordinating Centre. New ASPREE projects with appropriate scientific merit may be proposed by external researchers, and submitted to ASPREE for consideration. Project proposals requesting access to any aspect of data, samples, or analyses from the ASPREE clinical trial and/or sub-studies must gain the support of the ASPREE Principal Investigators. Applications are submitted via a secure web site, the ASPREE Access Management System (AMS). Applicants can obtain information by contacting aspree.ams@monash.edu.
