# Supplementary material for: Correlates of Meal Skipping in Community Dwelling Older Adults: A Cross-Sectional Study
Source: J Nutr Health Aging. Author manuscript; Available in PMC 2024 Jan 1. (PMC10035663; doi:10.1007/s12603-023-1884-2)
Supplement: 1 [file NIHMS1878845-supplement-1.docx]

Supplementary Table 1: Measurement of Correlates

| **Correlate** | **Measured** | **Measurement** |
| --- | --- | --- |
| Sex | Baseline Questionnaire | Male or female |
| Education Level | Baseline Questionnaire | Years of education |
| Geographic Residential Classification | Baseline Questionnaire | Major cities vs inner regional/outer regional remote |
| Index of Relative Social Advantage and Disadvantage (IRSAD) | Baseline Questionnaire | Quintile Score - area level advantage and disadvantage based on variables including; employment, occupation, income, education, house size and ownership and rental prices ^1^ |
| Polypharmacy | Baseline Questionnaire | Self-reported: medications use, with using 5 or more prescription medications defined as polypharmacy |
| Hypertension | Baseline Questionnaire | Systolic blood pressure: ≥140mmHg and diastolic blood pressure ≥ 90mmHg or taking blood pressure-lowering medication ^2, 3^ |
| Diabetes | Baseline Questionnaire | Self-reported, medicated for diabetes or a fasting glucose ≥126mg/dL ^2, 3^ |
| Frailty | Baseline Questionnaire | Modified Fried frailty criteria which included low body weight, weak grip strength, exhaustion, low physical activity and slow walking speed. ^3, 4^ Those classified as pre-frail met one or two of the Fried criteria, and those classified as frail met 3 or more criteria. ^3, 4^ |
| Smoking Status | Year 3 ASPREE Questionnaire | Self-reported: Smoker or non-smoker |
| Alcohol consumption | Year 3 ASPREE Questionnaire | Self-reported: Yes or No & Standard drinks per day:1, 2, 3, 4, >4 |
| Centre for Epidemiological Studies Depression (CES-D) scale | Year 3 ASPREE Questionnaire | A score 2 or less signifies no depressive symptoms, between 3 and 7 mild depressive symptoms and a score of 8 or above indicates presence of significant depressive symptoms |
| Quality of Life | Year 3 ASPREE Questionnaire | Mental Component Score (MCS) & Physical Component Score (PCS) generated from SF-12 Questionnaire |
| Reading food labels | Year 3 ALSOP Medical Questionnaire | Self-reported: Difficulty level – none, little, sometimes extreme |
| BMI | Year 3 ALSOP Medical Questionnaire | Weight and height by dividing weight in kilograms by height in meters squared. ^2, 3^ |
| Oral Health Status | Year 3 ALSOP Medical Questionnaire | Self-reported: excellent/very good, good/fair, poor |
| Saliva Levels | Year 3 ALSOP Medical Questionnaire | Self-reported: Over production, sufficient production, under production. |
| Pain Frequency | Year 3 ALSOP Medical Questionnaire | Self-reported: never, rarely (less than once a month), sometimes (1-3 times a month), often (once a week or more), always (most days). |
| Living Status | Year 3 ALSOP Social Questionnaire | Self-reported: lives alone, or with friend, family, spouse, in residential home or in supervised care”. |

References

1. Australian Bureau of Statistics. 2033.0.55.001 - Census of Population and Housing: Socio-Economic Indexes for Areas (SEIFA), Australia, 2011 <https://www.abs.gov.au/ausstats/abs@.nsf/Lookup/2033.0.55.001main+features100042011>. 2011;

2. McNeil JJ, Woods RL, Nelson MR, et al. Baseline Characteristics of Participants in the ASPREE (ASPirin in Reducing Events in the Elderly) Study. *The journals of gerontology Series A, Biological sciences and medical sciences*. 2017;72(11):1586-1593. doi:10.1093/gerona/glw342

3. McNeil JJ, Woods RL, Nelson MR, et al. Effect of Aspirin on Disability-free Survival in the Healthy Elderly. *New England Journal of Medicine*. 2018;379(16):1499-1508. doi:10.1056/NEJMoa1800722

4. Wolfe R, Murray AM, Woods RL, et al. The aspirin in reducing events in the elderly trial: Statistical analysis plan. *Int J Stroke*. 2018;13(3):335-338. doi:10.1177/1747493017741383
