# Supplementary material for: Correlates of Meal Skipping in Community Dwelling Older Adults: A Cross-Sectional Study
Source: J Nutr Health Aging. Author manuscript; Available in PMC 2024 Jan 1. (PMC10035663; doi:10.1007/s12603-023-1884-2)
Supplement: 2 [file NIHMS1878845-supplement-2.docx]

*Supplementary Table 2. The association of demographic, social, behavioural, biomedical, and psychological factors with meal skipping in adults aged 70 years and over: results of univariable and multivariable binary logistic regression analyses*

| Variable | Unadjusted OR (95% CI), p value  Univariable models | Adjusted OR (95% CI), p value  Multivariable model |
| --- | --- | --- |
| **Sex**  Male  Female | Ref  0.97 (0.88-1.07), 0.558 | Ref  0.84 (0.75-0.94), 0.003 |
| **Age**  70-74.99  75-79.99  80-84.99  85+ | Ref  0.89 (0.80-1.00), 0.050  0.80 (0.69-0.93), 0.004  0.71 (0.57-0.87), <0.001 | Ref  0.86 (0.76-0.96), 0.011  0.70 (0.60-0.82), <0.001  0.56 (0.45-0.70), <0.001 |
| **Education**  **=/<12 years**  >12 years | Ref  1.15 (1.04-1.28), 0.004 | Ref  1.15 (1.04-1.28), 0.007 |
| **Living Status**  With others  Alone | Ref  1.65 (1.50-1.83), <0.001 | Ref  1.84 (1.64-2.05), <0.001 |
| **Rurality**  Inner Cities  Regional | Ref  0.81 (0.73-0.89), <0.001 | Ref  0.81 (0.72-0.92), 0.001 |
| **IRSAD**  1 (most disadvantaged)  2  3  4  5 (least disadvantaged) | Ref  1.07 (0.89-1.30), 0.453  1.06 (0.89-1.26), 0.483  1.08 (0.91-1.28), 0.376  1.18 (1.00-1.40), 0.040 | Ref  1.09 (0.91-1.31), 0.340  1.05 (0.88-1.25), 0.578  0.99 (0.83-1.20), 0.994  1.05 (0.87-1.26), 0.623 |
| **Alcohol drinks per day**  0  1-2  3-4  Over 4 | Ref  1.10 (0.94-1.26), 0.149  1.25 (1.02-1.53), 0.025  2.18 (1.55-3.06), <0.001 | Ref  1.07 (0.93-1.23), 0.327  1.18 (0.98-1.45), 0.120  1.93 (1.35-2.75), <0.001 |
| **Smoking status**  Past/Non-smoker  Smoker | Ref  2.40 (1.78-3.15), <0.001 | 2.07 (1.54-2.80), <0.001 |
| **Hypertension**  No  Yes | Ref  0.98 (0.90-1.10), 0.827 | Ref  0.97 (0.86-1.10), 0.637 |
| **Diabetes**  No  Yes | Ref  1.30 (0.10-1.53), 0.002 | Ref  1.26 (1.06-1.50), 0.007 |
| **Frailty**  Not frail  Pre frail  Frail | Ref  1.03 (0.93-1.15), 0.465  1.93 (1.31-2.84), 0.001 | Ref  0.99 (0.89-1.10), 0.828  1.63 (1.09-2.43), 0.018 |
| **Polypharmacy**  No  Yes | Ref  1.80 (1.05-1.32), 0.005 | Ref  1.04 (0.92-1.18), 0.514 |
| **Pain**  Never  Rarely sometimes  Often/always | Ref  1.14 (0.96-1.36), 0.138  1.38 (1.15-1.65), <0.001 | Ref  1.04 (0.89-1.27), 0.513  1.19 (0.98-1.45), 0.072 |
| **Reading Labels**  No difficulty  Some difficulty  Difficulty | Ref  1.23 (1.11-1.36), <0.001  1.72 (1.35-2.20), <0.001 | Ref  1.14 (1.02-1.27), 0.013  1.44 (1.12-1.86), 0.005 |
| **Oral Health**  No difficulty  Some difficulty  Difficulty | Ref  1.34 (0.21-1.48), <0.001  2.26 (1.44-3.53), <0.001 | Ref  1.21 (1.10-1.35), <0.001  1.71 (1.07-2.73), 0.023 |
| **Saliva status**  Right amount  Too much  Too little | Ref  1.57 (1.25-1.99), <0.001  1.31 (1.07-1.60), 0.007 | Ref  1.38 (1.08-1.75), 0.008  1.09 (0.88-1.33), 0.436 |
| **MCS**  Under average  Over average | Ref  0.71 (0.64-0.80), <0.001 | Ref  0.76 (0.69-0.85), <0.001 |
| **PCS**  Under average  Over average | Ref  0.87 (0.80-0.96), 0.005 | Ref  0.93 (0.83-1.04), 0.271 |

*IRSAD=Index of Relative Socioeconomic Advantage and Disadvantage ^1^ PCS= Physical component scores of the SF-12 questionnaire, MCS= Mental Component Score of the SF-12 questionnaire*

References

1. Australian Bureau of Statistics. 2033.0.55.001 - Census of Population and Housing: Socio-Economic Indexes for Areas (SEIFA), Australia, 2011 <https://www.abs.gov.au/ausstats/abs@.nsf/Lookup/2033.0.55.001main+features100042011>. 2011;
